# Characterization of the Proinflammatory Profile of Synovial Fluid-Derived Exosomes of Patients with Osteoarthritis

**DOI:** 10.1155/2017/4814987

**Published:** 2017-05-28

**Authors:** Rossana Domenis, Rossella Zanutel, Federica Caponnetto, Barbara Toffoletto, Adriana Cifù, Cinzia Pistis, Paolo Di Benedetto, Araldo Causero, Massimo Pozzi, Fabrizio Bassini, Martina Fabris, Kayvan R. Niazi, Patrick Soon-Shiong, Francesco Curcio

**Affiliations:** ^1^Dipartimento di Area Medica (DAME), Università degli Studi di Udine, Udine, Italy; ^2^Clinica Ortopedica, Azienda Sanitaria Universitaria Integrata di Udine, Udine, Italy; ^3^Dipartimento di Ortopedia e Traumatologia, Azienda per l'Assistenza Sanitaria n.3 Alto Friuli, Tolmezzo, Italy; ^4^Istituto di Patologia Clinica, Azienda Sanitaria Universitaria Integrata di Udine, Udine, Italy; ^5^NantBioScience, Inc., Culver City, CA 90232, USA

## Abstract

The purpose of this study is to characterize synovial fluid- (SF-) derived exosomes of patients with gonarthrosis comparing two methods of isolation and to investigate their immune regulatory properties. Extracellular vesicles (EVs) have been isolated from inflamed SF by polymer precipitation method and quantified by Exocet kit and by nanoparticle tracking analysis. Vesicles expressed all the specific exosomal markers by immunoblot and FACS. After isolation with Exoquick, a relevant contamination by immune complexes was detected, which required further magnetic bead-based purification to remove. SF-derived exosomes significantly stimulated the release of several inflammatory cytokines and chemokines and metalloproteinases by M1 macrophages but did not influence the expression of CD80 and CD86 costimulatory molecules. In conclusion, we characterized purified exosomes isolated from inflamed SF and demonstrate that purified exosomes are functionally active in their ability to stimulate the release of proinflammatory factors from M1 macrophages. Our data indicate that SF-derived exosomes from gonarthrosis patients play a role in disease progression.

## 1. Introduction

Osteoarthritis (OA) is a chronic and progressive musculoskeletal disorder that involves the entire synovial joint and affects patient quality of life. Although multiple pathophysiological mechanisms are involved, the activation of the innate and immune systems resulting in inflammation is a key component in promoting synovitis as well as progression of cartilage and bone destruction [1].

Previous studies investigating the inflammatory cells involved in OA reported that macrophages and T lymphocytes are the most abundant cells, although mast cells and B cells were also found [2]. Histological studies demonstrated that synovial macrophages were mostly distributed in the lining layer and were activated in the inflamed joint [3]. Through the production of proinflammatory cytokines, growth factors, and enzymes, macrophages play a major role in inflammation, including the stimulation of angiogenesis, leukocyte and lymphocyte recruitment, fibroblast proliferation, and protease secretion leading to joint destruction [4, 5].

Exosomes are small membrane-bound extracellular vesicles (EVs) varying in size from 30 to 100 nm originating from internal budding of the plasma membrane during endocytic internalization. Exosomes are released from normal and pathological cells and are present in blood and other bodily fluids, including malignant ascites, urine, amniotic and synovial fluids, and saliva [6]. The cargo of exosomes (which can include proteins, messenger and microRNAs, lipids, and metabolites) reflects the biological state of the parent cells and can be transferred to others cells, acting in a paracrine or even an endocrine manner to modify the behaviour of adjacent or distant cells [7].

Recently, it has been proposed that EVs could play a role in the pathogenesis of numerous inflammatory conditions. EVs (carrying pathogen-associated damage-associated molecular patterns, cytokines, autoantigens, and tissue-degrading enzyme) create a microenvironment that triggers inflammation and sustains the progression of the disease [8]. Synovial fluid- (SF-) derived EVs remain poorly characterized to date. Citrullinated proteins, known to be autoantigens in rheumatoid arthritis (RA), were detected in exosomes purified from SF of RA patients [9]. Moreover, it has been described that microvesicles derived from CD4+ T cells, CD19+ B cells, and CD14+ monocytes are present in OA, RA, and juvenile idiopathic arthritis SF [10].

Several studies have demonstrated that EVs, produced in inflamed joints, may contribute to disease progression. Exosomes released from synovial fibroblasts obtained from RA patients were found to contain a membrane form of TNF*α* that makes activated T cells resistant to apoptosis, favouring the pathogenic process of RA [11]. In addition, microvesicles derived from activated monocytes and T cells induce the synthesis of metalloproteinases (MMPs) [12] and cytokines/chemokines [13] in RA and OA synovial fibroblasts. In turn, exosomes from activated synovial fibroblasts induce osteoarthritic changes in articular chondrocytes [14].

Despite these earlier findings, the immune regulatory properties of the exosomes isolated from inflamed SF have never been investigated. In this study, we characterized SF-derived exosomes isolated from inflamed joints of patients with gonarthrosis using an integrated system. We compared two methods of exosome isolation in order to identify the least contaminated preparation. We then evaluated the immune regulatory capacity of purified exosomes on M1 macrophages, since these cells have a key role in the pathogenesis of OA.

## 2. Methods

### 2.1. Isolation of Exosomes from Inflamed Synovial Fluid

Patients scheduled for first-intention knee replacement surgery due to end-stage knee osteoarthrosis were recruited at the Orthopedic Units of the Hospital of Udine and of the Hospital of Tolmezzo. All patients gave written informed consent.

Inflamed synovial fluids (SF) were obtained by needle aspiration from *n* = 10 patients (4 males and 6 females, 72 ± 8 years). To clarify samples, SF were treated with 2 mg/ml bovine testicular hyaluronidase type I-S (Sigma-Aldrich) for 30 minutes, then centrifuged at 14,000*g* for 20 minutes, and supernatants were stored at −80°C until use.

To isolate EVs, SF was incubated with ExoQuick-TC (System Biosciences) overnight at 4°C and was then centrifuged twice at 1500*g* for 30 and 5 minutes, respectively. The EV-containing pellet was then resuspended in PBS buffer or lysis buffer for subsequent analysis. This technology works by capturing and collecting EVs in “polymer nets,” which can be recovered by low-speed centrifugation. Once the EV-containing pellet is obtained, the supernatant with the polymer in excess is removed and the EVs can be resuspended in a suitable solution, dissolving the polymer net and releasing intact exosomes [15].

In order to evaluate their immune regulatory properties, exosomes have been purified by immunoaffinity using Tetraspanin Exo-Flow combo capture kit (System Biosciences), according to the manufacturer's instructions. Briefly, streptavidin-conjugated magnetic beads [9.1 *μ*m, 1.6 × 10^7^ beads/ml] were coupled with anti-CD9, anti-CD63, and anti-CD81 biotinylated antibody for 2 h on ice. Afterwards, exosomes were incubated on a rotating rack at 4°C overnight. To validate the isolation procedure, exosome-coated beads were stained on ice for 2 h with Exo-FITC exosome stain (System BioScience) and then analysed on a FACSCalibur flow cytometer (BD Biosciences). Finally, exosomes were eluted from magnetic beads using the elution buffer for 2 h on a rotating rack. Contamination by immune complexes in isolated exosomes was evaluated by immunofixation diagnostic assay performed on the fully automated gel electrophoresis instrument InterlabG26 (Interlab).

### 2.2. Exosome Validation

#### 2.2.1. Quantification and Size Profiling

The number of SF-derived exosomes was determined using the Exocet kit (System Biosciences), according to the manufacturer's instructions. The exosomes were lysed using a gentle lysis solution as to preserve the enzymatic activity of the exosomal Acetylcholinesterase (AChE) enzyme. A standard curve was generated using known numbers of exosomes (as measured by NanoSight) and calibrated with a recombinant AChE enzyme standard solution provided in the kit.

The number and size of purified exosomes were determined by nanoparticle tracking analysis (NTA) equipped with a 532 nm laser (Nanosight LM10 system, Malvern Instrument Ltd.) that tracks the particles' Brownian motion estimating the particle size and concentration. To do this, 500 *μ*l of an appropriately diluted (1 : 400) exosome-enriched preparation was used, and for each sample, a 60 s video was captured at a fixed detection threshold of 16. Temperature was monitored throughout the measurements. Vesicle size distribution and an approximate concentration were obtained from the raw data displayed by the associated software. The experiments were conducted in triplicate.

#### 2.2.2. Flow Cytometry

One milligram of aldehyde/sulfate latex beads (4 *μ*m, Invitrogen) was incubated with 5 *μ*g of purified anti-CD63 antibody (BD Pharmingen) under agitation overnight at 4°C. SF-derived EVs (1 × 10^10^) were incubated with 5 *μ*g anti-CD63 beads in 100 *μ*l PBS for 15 min at room temperature; the volume was made up to 300 *μ*l and incubated for 2 hours at room temperature on a rotator. To block free binding sites, EV-coated beads were incubated for 30 minutes with 200 mM glycine. After two washes in PBS with 0.1% BSA, EV-coated beads were stained with the primary antibody anti-CD9 Alexa 647 (Serotec), anti-CD81 FITC (Biolegend), and anti-CD7 PE (Becton Dickinson) or isotype control (BD Biosciences) and analysed using a FACSCalibur flow cytometer (BD Biosciences).

Exosomes purified by immunoaffinity Exo-Flow kit were stained with specific monoclonal antibodies anti-CD81 FITC (Biolegend), anti-CD63 FITC (Santa Cruz), anti-CD9 PE (eBiosciences), anti-CD61 FITC (BD Biosciences), and anti-CD41a FITC (BD Biosciences) and analysed by flow cytometry.

#### 2.2.3. Immunoblotting

Isolated exosomes were lysed in RIPA buffer (150 mM sodium chloride, 1% NP-40, 0.5% sodium deoxycholate, 0.1% SDS, 50 mM Tris, pH 8.0) supplemented with protease inhibitors (Sigma-Aldrich) for 30 minutes on ice. Immunoglobulins were purified from preparations of EVs with IgG Pure Proteome™ protein A magnetic beads kit (Millipore), according to the manufacturer's instructions.

The lysate was quantified by Bradford assay (Bio-Rad Laboratories), and 25 *μ*g of proteins derived from EVs isolated by polymer precipitation, 2 *μ*g for exosomes isolated by immunoaffinity, and 5 *μ*g of immunoglobulins were mixed with 4× sample buffer (8% SDS, 20% 2-mercaptoethanol, 40% glycerol, 0.008% Bromophenol blue, 0.25 M Tris, pH 6.8) and boiled for 10 minutes at 95°C. Proteins were resolved by SDS-PAGE, transferred to PVDF membranes, blocked in 5% nonfat powdered milk or BSA in TBS-T (20 mM Tris pH 7.5, 150 mM NaCl, 0.1% Tween-20), and stained with the following antihuman antibodies: anti-CD9 (1 : 1000, System BioScience), anti-CD63 (1 : 1000, LS Bio), anti-CD81 (1 : 500, Abcam), anti-TSG101 (1 : 500, Abcam), anti-RISC complex (1 : 1000 Abcam), anti-calnexin (1 : 1000 Enzo Life Technologies), and anti-58K (1 : 1000 Genetex). To visualize binding, horseradish peroxidase-conjugated secondary antibodies (1 : 1000, Dako) were used (ECL western blotting substrate, Thermo Scientific).

### 2.3. Monocyte Isolation and Differentiation into M1 Macrophages

Human peripheral blood mononuclear cells (PBMCs) were isolated from EDTA-uncoagulated blood of blood donors by Ficoll gradient centrifugation (Millipore). Monocytes were separated from PBMCs by negative selection using a human CD14+ cell enrichment kit (StemCell Technologies) according to the manufacturer's instructions and resuspended in RPMI medium supplemented with 10% heat inactivated fetal bovine serum (FBS), 1% glutamine, 1% pyruvate, 1% nonessential aminoacid, 1% penicillin/streptomycin, 1% Hepes (all from Euroclone). To remove the exosomal fraction present in FBS, serum was ultracentrifuged for 4 hours at 100,000*g*. Purity of monocytes was over 95% as judged by staining with anti-CD14 (eBiosciences) and flow cytometry analysis (FACSCalibur). For macrophage differentiation, CD14+ monocytes were seeded in multiwell plates at 5 × 10^5^/cm^2^ in complete RPMI medium supplemented with 100 ng/ml granulocyte macrophage colony-stimulating factor (GM-CSF, Peprotech) and cultivated for 10 days. Medium was changed completely every 3 days.

### 2.4. Gene Expression Analysis of M1 Macrophages

M1 macrophages (3 × 10^5^ cells) were incubated with 7.5 × 10^9^ SF-derived exosomes for 6 h, and RNA was extracted using the RNeasy Mini kit (Qiagen). Purified RNA concentrations were determined spectrophotometrically using a NanoDrop ND100 spectrophotometer (ThermoScientific). Complementary DNA (cDNA) synthesis was performed using the iScript™ cDNA Synthesis Kit (Bio-Rad Laboratories) according to the manufacturer's protocol. Quantitative real-time polymerase chain reaction (RT-PCR) was performed using the Roche LightCycler 480 Real-Time PCR System utilizing SYBR® Green Master Mix (Bio-Rad Laboratories), following the manufacturer's instructions. The primers were designed from available human sequences using the primer analysis software Primer3. The sequences of the primers used in this study are the following:

TNF*α*: Fw:CCCATGTTGTAGCAAACCCT, Rv:TGAGGTACAGGCCCTCTGAT;

IL10: Fw:GCGCTGTCATCGATTTCTTCCC, Rv:GTTTCTCAAGGGGCTGGGTC;

IL6: Fw:ACATCCTCGACGGCATCTCA, Rv:TCACCAGGCAAGTCTCCTCATT;

IL1*β*: Fw:TGCCCGTCTTCCTGGGAGGG, Rv:GGCTGGGGATTGGCCCTG.

GAPDH: Fw:AGTATGACAACAGCCTCAAG, Rv:TCTAGACGGCAGGTCAGGTCCAC.

The expression level of each gene was assessed as compared to the expression of GAPDH. The ΔΔCt method was used for analysis of RT-PCR data.

### 2.5. Analysis of CD80/CD86 Expression Level and Cytokine/Chemokine/MMP Secretion by M1 Macrophages

M1 macrophages (3 × 10^5^) were incubated with 7.5 × 10^9^ SF-derived exosomes for 24 hours or stimulated with 150 UI/ml IFN*γ* (Peprotech) for 12 hours and then with 10 ng/ml LPS (Sigma-Aldrich) for 24 hours. The stimulation with IFN*γ*/LPS was used as a positive control for these experiments. The expression level of the costimulatory molecules CD80 and CD86 was evaluated by flow cytometry using the anti-CD80 (eBiosciences) and anti-CD86 (eBiosciences) antibodies. Flow cytometry was carried out on the FACSCalibur (Becton Dickson) and data analysed using Flowing software. Supernatants were also harvested, centrifuged for 10 minutes at 14,000*g*, and cytokine/chemokine and MMP concentrations were quantified with a magnetic bead-based multiplex assay (Bio-plex Assay, Bio-Rad Laboratories). To ensure that cytokines were not previously present in our exosome preparation as contaminants, their presence was investigated directly in SF-derived isolated exosomes by magnetic bead-based multiplex assay.

### 2.6. Statistical Methods

Data are reported as mean ± standard deviation. Statistical analysis has been performed using GraphPad Software (version 4.0c). Data were tested for normal distribution using the Kolmogorov-Smirnov test. Paired *t*-test or Wilcoxon test, as appropriate, was used to compare continuous variables between two groups. A *p* value less than 0.05 was considered significant.

## 3. Results

### 3.1. Characterization of SF-Derived EVs Isolated by the Polymer Precipitation Method

**Figure 1:**
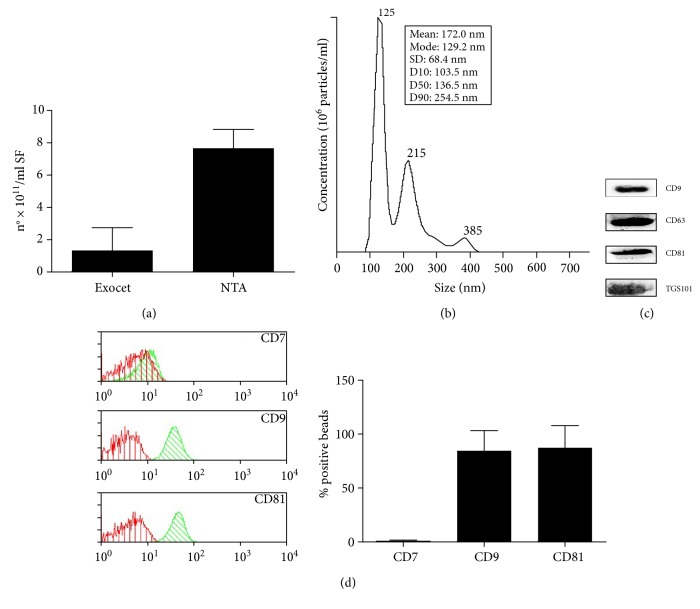
Characterization of SF-derived EVs isolated by Exoquick polymer precipitation. (a) Particle concentration was quantified measuring the enzymatic activity of the exosomal AChE enzyme by Exocet kit or tracking the particles' Brownian motion by Nanosight NTA. Columns, mean; bars, SD. (b) A representative particle size profiling by NTA is shown. (c) SF-derived EVs were lysed, and 25 *μ*g of protein was separated in 15% SDS-PAGE gel under reducing condition. The gel was western blotted onto nitrocellulose membranes and stained with antibodies recognizing exosomal marker proteins CD9, CD63, CD81, and TSG101. (d) EVs were embedded into 4 *μ*m beads coated with anti-CD63 and then stained with specific monoclonal antibody for CD7, CD9, and CD81 and analysed by flow cytometry. The antibodies (green peak) were compared with their appropriate isotype control (red peak). Cytometry histograms are shown as one representative experiment. The histograms represent the percentages of CD7-, CD9-, and CD81-positive beads. Columns, mean; bars, SD.

The concentration of EVs isolated by the polymer precipitation method was determined either measuring the activity of AChE by Exocet kit or by using nanoparticle tracking analysis (NTA), and the two results were compared (Figure 1(a)). The mean concentration estimated by NTA was higher (7.6 ± 3.1 × 10^11^ particles/ml) compared to that measured by Exocet kit (1.3 ± 1.4 × 10^11^ EVs/ml). As shown in the representative report of particle size profiling measured by NTA (Figure 1(b)), the majority of the particles were within the range size of 100 nm (higher peak), but two other lower peaks corresponding to microvesicles with larger sizes were also apparent.

To validate the polymer precipitation method, we analysed the expression of specific exosomal markers by immunoblot analysis. As shown in (Figure 1(c)), the isolated SF-derived EVs expressed the surface tetraspanins CD9, CD63, CD81, and the internal marker TSG101, indicating the validity of this method to isolate exosomes from the synovial fluid. Images of the entire immunoblots are available as Supplementary Figure 1 available online at https://doi.org/10.1155/2017/4814987. The appearance of multiple bands is due to the presence of human immunoglobulins, which coprecipitate with EVs and bound HRP-conjugated secondary antibodies. In order to confirm the specificity of the signals of exosomal markers, we purified immunoglobulins from the preparations of EVs and analysed them together with sample of EVs by immunoblotting. The comparison between EVs and IgG blots showed the presence of the specific bands related to the exosomal markers under investigation.

The expression of CD81 and CD9 was confirmed also by flow cytometry analysis on exosome-coated CD63 beads (Figure 1(d)). The majority of CD63+-positive exosomes expressed CD9 (84.6 ± 18.8%) and CD81 (87.3 ± 20.6%). The antibody for CD7 was used as negative control to validate the specificity of the assay: the lack of CD7+ exosomes demonstrates the absence of nonspecific binding of antibodies to the beads.

### 3.2. Characterization of SF-Derived Exosomes Isolated by the Immunoaffinity Purification Method

**Figure 2:**
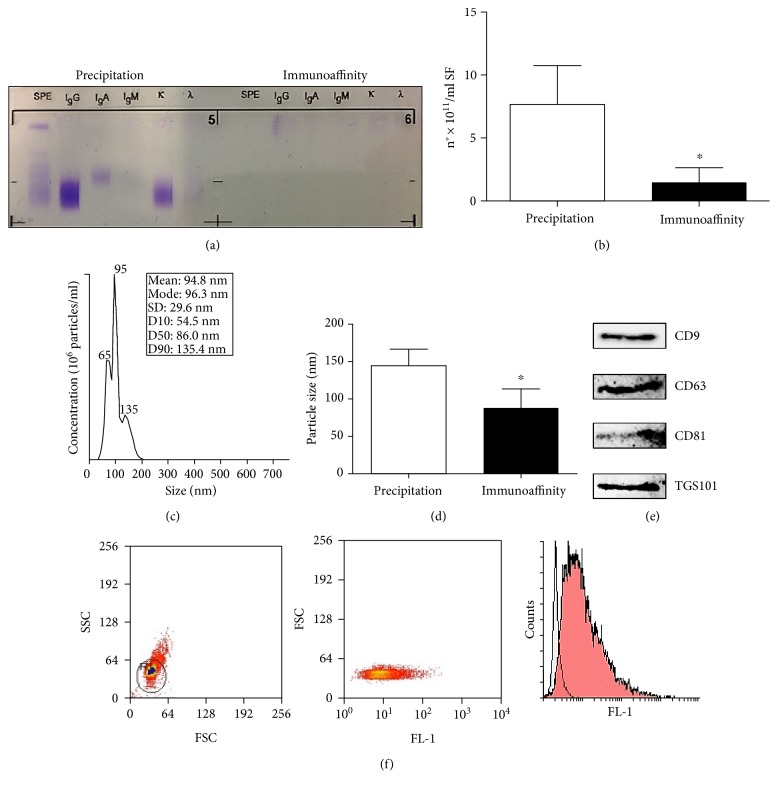
Characterization of SF-derived exosomes isolated by immunoaffinity. (a) SF-derived exosomes isolated by Exoquick polymer precipitation or immunoaffinity magnetic beads were assessed for immune complex contamination by immunofixation. The immunofixation gel is shown as one representative experiment. The particle concentration (b) and size (d) were quantified tracking the particles' Brownian motion by Nanosight NTA. Columns, mean; bars, SD, ^∗^significant difference *p* < 0.05. (c) A representative particle size profiling by NTA is shown. (e) SF-derived exosomes were lysed, and 2 *μ*g of protein was separated in 15% SDS-PAGE gel under reducing condition. The gel was blotted onto nitrocellulose membranes and stained with antibodies against exosomal markers CD9, CD63, CD81, and TSG101. (f) Exosomes were bound by Exo-Flow beads, stained with Exo-FITC, and analysed by flow cytometry. Histograms of one representative experiment are shown. Exosome-bound beads (red peak) were compared with beads alone (white peak).

It has been demonstrated that polymer precipitation of exosomes can result in contamination by protein aggregates that copurify with nanovesicles [16]; in particular, the biophysical properties of immune complexes overlap with microvesicles, interfering with the interpretation of ensuing results [17]. The experimental results described in this manuscript confirmed the presence of immune complex contamination in purified vesicle preparations (Figure 2(a), left panel). Considering that immune complexes may have important immune regulatory activities in functional studies, exosome extracts were further purified using immunomagnetic beads. As illustrated in the right panel of (Figure 2(a)), exosomes purified by immunoaffinity were not contaminated by immune complexes. Size and concentration of SF-derived exosomes purified by immunoaffinity were then determined by NTA analysis, and results were compared with data obtained on exosomes isolated by polymer precipitation method. We observed that the concentration of the particles purified by immunoaffinity (1.5 ± 1.2 × 10^11^ particles/ml) was significantly lower compared to the concentration measured on the same samples purified by polymer precipitation (7.6 ± 3.1 × 10^11^ particles/ml) (Figure 2(b)). As shown in the representative report of particle size profiling measured by NTA (Figure 2(c)), all the microparticles were approximately 100 nm in size. These data confirmed the presence of nonexosomal contaminants in exosome samples isolated by polymer precipitation, which corresponds to the peaks comprise within 200 and 300 nm in (Figure 1(a)). As expected, the size distribution of the particles purified by the immunoaffinity method (88.01 ± 25.5 nm, range: 55.5–125.9 nm) was significantly more restricted compared to the particles purified by polymer precipitation (144.4 ± 22.2 nm, range 124–187.9 nm) (Figure 2(d)).

To confirm the integrity of exosomes isolated by immunoaffinity, we assessed the expression of CD9, CD63, CD81, and TSG101 by immunoblotting (Figure 2(e)). Images showing the entire blotted membranes are available as Supplementary materials (see Figure 2). The appearance of multiple bands is due to the presence of the antibodies (heavy and light chains) used to capture exosomes. In fact, when ExoFlow elution buffer is used, the exosomes and capture antibodies are both released from the beads. Therefore, to confirm the specificity of the signals of exosomal markers, we purified immunoglobulins from exosome preparations and analysed them by immunoblotting together with the corresponding samples of exosomes. The comparison between exosome and IgG blots showed the presence of bands specific for the exosomal markers under investigation.

In order to evaluate the impurities in our exosome preparations, we evaluated the expression of proteins associated with subcellular compartments, which are supposed to be absent or underrepresented in exosomes, by immunoblotting analysis. The expression of calnexin (from endoplasmic reticulum), 58K (from Golgi), and RISC complex (from nucleus) in SF-derived exosomes was evaluated. The results are reported in Supplementary material section (Figure 2) and indicate that no contaminants were present in exosomes purified by immunoaffinity.

Moreover, exosome isolation was further confirmed by staining vesicles with ExoFITC, a dye that binds posttranslational modifications of surface protein (such as glycosylation). As shown in Figure 2(f), 88.6 ± 7.2% of the exosomes bound to the beads reacted with the ExoFITC reagent. Finally, in order to evaluate whether SF-derived exosomes were of platelet origin, we analysed the expression of platelet markers (CD41a and CD61) on the surface of exosomes purified by flow cytometry. The results (Supplementary Figure 3) show that exosomes isolated by immunoaffinity purification did not express platelet markers, instead expressed CD9, CD81, and CD63 exosomal markers, as expected.

### 3.3. Proinflammatory Effects of SF-Derived Exosomes on M1 Macrophages

**Figure 3:**
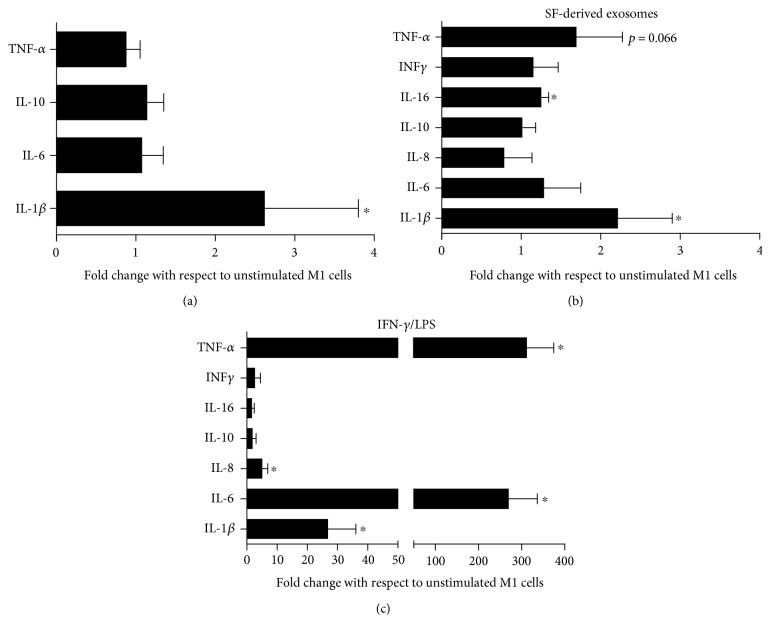
SF-derived exosomes stimulate the production of several cytokines by M1 macrophages. (a) M1 macrophages, differentiated from normal donor monocytes, were incubated with SF-derived exosomes for 6 hours and cytokine expression was analysed by RT-PCR. Data were expressed as fold change relative to unstimulated cells. (b) M1 macrophages were treated with SF-derived exosomes (b) or IFN-*γ*/LPS (c) for 24 hours, and cytokine production was quantified in supernatants by ELISA Bio-plex cytokine assay system. Data were expressed as fold change relative to unstimulated cells. Columns, mean; bars, SD, ^∗^significant difference from unstimulated cells *p* < 0.05.

At the transcriptional level, M1 macrophages stimulated with SF-derived exosomes displayed a significant upregulation of IL-1*β* expression, while mRNA levels of other cytokines TNF*α*, IL10, and IL6 were not changed (Figure 3(a)).

These results were confirmed at the protein level, since evaluation of a large panel of proinflammatory cytokines by multiplex technology demonstrated that M1 macrophages treated with SF-derived exosomes (Figure 3(b)) release significant amounts of IL-1*β* and to a lesser extent IL-16, but not TNF-*α* and IL-6. Of note, IL-8 secretion was decreased. In contrast to the profile of cytokines released in response to INF-*γ*/LPS coincubation, SF-derived exosomes stimulate the release of IL-6 and TNF-*α*, followed by a much lower production of IL-1*β* and IL-8 (Figure 3(c)). IL-10 production was substantially unaffected, confirming the M1 phenotype of the macrophages.

**Figure 4:**
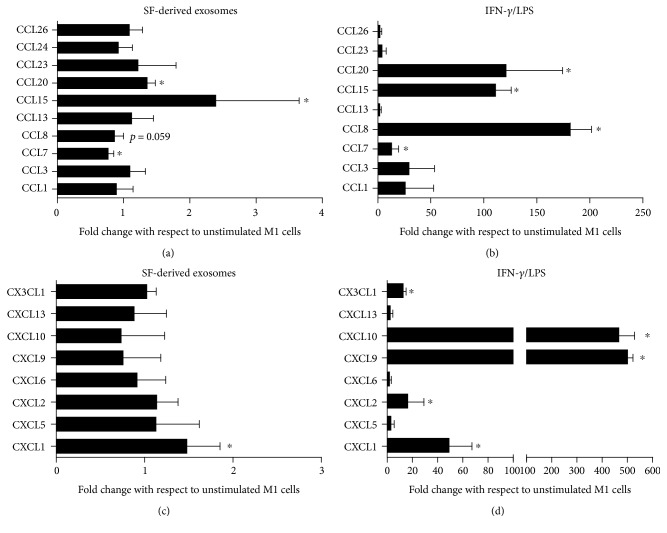
SF-derived exosomes stimulate the production of several chemokines by M1 macrophages. M1 macrophages were treated with SF-derived exosomes (a–c) or IFN-*γ*/LPS (b–d) for 24 h, and chemokine production in supernatants was determined by the ELISA Bio-plex chemokines assay system. Data are displayed as fold change with respect to unstimulated cells. Columns, mean; bars, SD, ^∗^significant difference from unstimulated cells *p* < 0.05.

In a similar manner, SF-derived exosomes induced the release of several chemokines (CCL8, CCL15, CCL20, and CXCL1), while downregulating the production of others (CCL7 in particular; Figures 4(a) and 4(c)). These results differed significantly from those observed after treatment with IFN*γ*/LPS, which stimulate the release of a broader spectrum of chemokines (Figures 4(b) and 4(d)).

**Figure 5:**
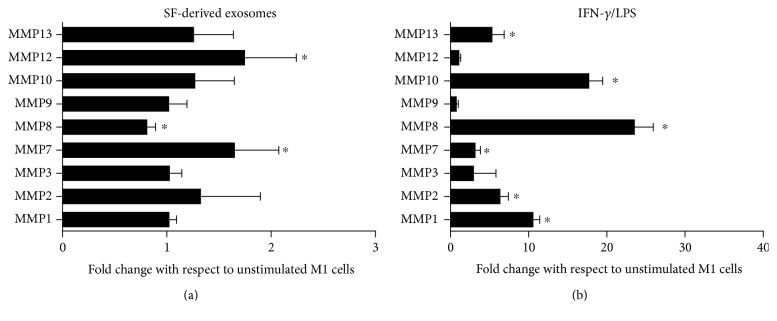
SF-derived exosomes stimulate the production of several MMPs by M1 macrophages. M1 macrophages were treated with SF-derived exosomes (a) or IFN-*γ*/LPS (b) for 24 h, and MMP production was determined in supernatants by the ELISA Bio-plex assay system. Data are expressed as fold change in relation to unstimulated cells. Columns, mean; bars, SD, ^∗^significant difference from unstimulated cells *p* < 0.05.

Finally, the production of MMPs by M1 macrophages treated with SF-derived exosomes was also evaluated. Treatment of M1 macrophages with SF-derived exosomes results in the release of MMP12 and MMP7 and the inhibition of MMP8 production (Figure 5(a)). In contrast, the incubation with IFN-*γ*/LPS stimulated the production of a wide assortment of MMPs (MMP1, MMP2, MMP7, MMP8, MMP10, and MMP13), but did not affect MMP12 secretion (Figure 5(b)).

To ensure that the cytokines measured were not contaminants of the exosome preparations themselves, their presence was investigated directly in SF-derived purified exosomes. The results indicate that IL-1*β*, IL-16, and TNF-*α* were below the level of detection in these preparations (data not shown).

**Figure 6:**
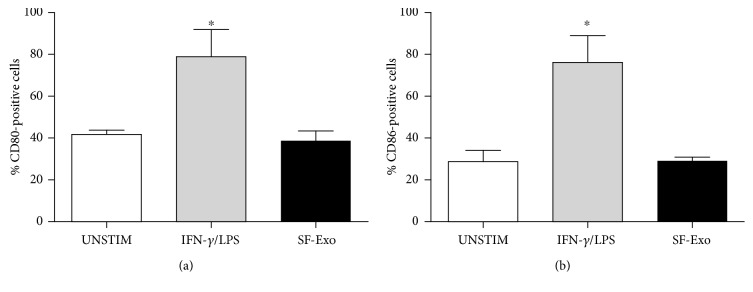
Expression of CD80 and CD86 by M1 macrophages following exposure to SF-derived exosomes. M1 macrophages were incubated in the presence of IFN-*γ*/LPS (grey column) or SF-derived exosomes (black column), and the percentage of CD80- (a) and CD86- (b) positive cells was determined by flow cytometry after 24 hours. Columns, mean; bars, SD. ^∗^significant difference from unstimulated cells *p* < 0.05.

It is well known that activated M1 macrophages upregulate the expression of CD80 and CD86, which are costimulatory molecules involved in T cell activation. Comparison of stimulation by SF-derived exosomes with control stimulation demonstrated that in contrast to IFN*γ*/LPS, SF-derived exosome treatment of M1 macrophages did not change significantly the expression of these costimulatory molecules (Figures 6(a) and 6(b)).

## 4. Discussion

Cell-derived extracellular vesicles (EVs) released in the synovial fluid (SF) of inflamed joints of patients with OA and RA are suggested to play a significant role in disease progression, triggering and contributing to the propagation of the inflammatory process and participating in tissue degeneration [8, 18]. It has been shown that EVs isolated from patient SF contain numerous autoantigens implicated in autoimmune disease [19–21] and can induce the release of proinflammatory cytokines and growth factors from synoviocytes in vitro [13, 22, 23].

There has been renewed interest in the use of EVs as biomarkers to monitor physiological and pathological processes. However, SF-derived EVs remain poorly characterized. Earlier studies by Skriner et al. [9] utilized electron microscopy and proteomic analysis to characterize isolated SF exosomes from patients with RA, highlighting the presence of fundamental RA autoantigens (i.e., citrullinated proteins) within such vesicles. In our study, we provide the first comprehensive characterization of SF-derived exosomes isolated from inflamed joints of patients with knee OA.

Towards this goal, two different isolation methods (based on polymer precipitation and immunoaffinity) were compared to identify an optimum means to purify exosomes from SF for downstream functional and biomarker studies. Previous studies using isolated exosomes have provided difficult to interpret results, making cross-comparisons between different studies difficult [15, 16]. Although differential centrifugation coupled with ultracentrifugation has been the most widely adopted method to isolate exosomes, it has also been suggested that ultracentrifugation can damage isolated vesicles, reducing their quality and thereby potentially impacting functional studies [16].

Our results demonstrated a discrepancy between the quantity of polymer-precipitated Exocet-quantified EVs and those counted by NTA, leading us to hypothesize the presence of contamination by other subcellular materials, such as immune complexes, lipoproteins, and protein aggregates. Further analysis identified immune complexes and protein aggregates as significant contributors to the contamination in SF fluid-derived EVs [10, 17] which could alter the validity of downstream functional assays and proteomic/genomic studies [15, 16, 24]. By incorporating an additional purification step utilizing immunoaffinity, the EV preparation was significantly enriched in exosomes. As reported for exosomes isolated from other biological samples (for details see http://microvesicles.org), we provide the first evidence that SF-derived exosomes express all the specific exosomal markers (CD9, CD63, CD81, and TSG101) and can be quantified through the determination of AchE activity.

Inflammation has been considered as the key player promoting synovitis as well as progression of cartilage and bone destruction in osteoarthritis (OA) [1]. It has been reported that macrophages become activated in the inflamed joint, where they account for about 30–40% of the cellular content and regulate secretion of proinflammatory cytokines and enzymes driving the inflammatory response [4]. Currently, several independent groups are attempting to identify and characterize the factors responsible for the development of inflammatory processes involved in OA. An analysis of the ever increasing number of reports directs attention to the special role of the cytokine network in the pathogenesis of OA, demonstrating that the production of cytokines can vary depending on the duration and severity of OA [25].

In our study, we investigated the immune regulatory properties of SF-derived exosomes on proinflammatory M1 macrophages differentiated from PBMCs of blood donors. These cells produced a spectrum of anticipated proinflammatory cytokines/chemokines and MMPs in response to our control stimuli. More importantly, these cells were activated by SF-derived exosomes to produce IL-1*β* and IL-16, supporting our hypothesis that SF-derived exosomes are able to sustain join inflammation acting on immune cells recruited in the synovium.

The key role of the proinflammatory IL-1*β* in the pathogenesis of OA is widely recognized. The levels of IL-1*β* are generally elevated in OA synovial fluid, indicating its possible importance in OA pathogenesis and progression [26] and making it a potential candidate as a biochemical marker [27, 28]. Importantly, IL-1*β* is upstream in the cascade of cytokines involved in OA, since it induces the expression and release of several other proinflammatory cytokines such as IL-8 and IL-6, as well as the synthesis of proteases including matrix metalloproteinases [29].

Interestingly, the role of IL-16, a proinflammatory cytokine with chemotactic activity towards CD4+ T lymphocytes, monocytes, and eosinophils, has never been thoroughly investigated. Recently, it has been reported that expression of IL-16 is decreased during chondrogenesis, whereas a marked increase was observed during OA progression [30].

In our study, we also observed that SF-derived exosomes are able to stimulate the production of CCL20, CCL15, and CXCL1 chemokines by M1 macrophages. Chemokines play a key role in the perpetuation of inflammation by attracting proinflammatory cells to the inflamed joint [31]. CCL20 may play an important role in the pathogenesis of OA by inducing changes in phenotype and catabolic gene expression in chondrocytes [32]. On the other hand, CXCL1 is upregulated in OA chondrocytes [33] and its activity is linked to cartilage development, where it induces chondrocyte hypertrophy and apoptosis [34, 35].

We also demonstrate that M1 macrophages stimulated by SF-derived exosomes release MMP12 and MMP7. Degradation of the cartilage extracellular matrix is a central feature of the OA and is widely thought to be mediated by proteinases that degrade structural components of the matrix, primarily aggrecan and collagen; MMPs are the primary enzymes responsible for the degradation of cartilage [36]. Notably, we observed that SF-derived exosomes, unlike IFN-*γ*/LPS, are able to promote the production of MMP12, suggesting that the vesicles act through a specific and alternative pathway compared to that involving LPS recognition (which are CD14/TLR4 mediated) or IFN*γ* signalling cascade. A recent study showed that the expression of MMP12 in the cartilage and subchondral bone of patients with OA correlates with disease severity, consistent with the findings described above [37]. On the other hand, a study conducted by Ohta and colleagues reported that MMP7 is overproduced in human OA cartilage and may play a significant role in the ECM degradation [38].

Finally, we report that SF-derived exosomes do not influence the expression of CD80 and CD86 costimulatory molecules on M1 macrophages, suggesting that they are not involved in the activation of T cells through this pathway.

In conclusion, we showed that exosomes isolated from the SF of inflamed joints of patients with OA are able to stimulate M1 macrophages to release key molecules involved in the inflammatory process and cartilage degeneration. Many studies have dealt with the immunosuppressive properties of exosomes, especially in oncology, while their proinflammatory effects are still poorly understood despite the considerable level of interest to better understand the mechanisms driving chronic inflammatory disorders.

Our data suggest that SF-derived exosomes may be key mediators in OA and could be a useful tool to recreate the inflammatory microenvironment typically associated with this disease in vitro. Our results provide a better understanding of the molecular and cellular mechanisms involved in the disease pathogenesis of gonarthrosis and lead the way for the identification of new and more effective therapeutic strategies.

## Supplementary Material

Figure 1. Immunoblotting analysis of SF-derived EVs. EVs isolated by precipitation were lysed and the protein fraction underwent SDS-PAGE gel electrophoresis under reducing condition. The IgG fraction was run together with the sample from which they have been extracted. The gel was blotted onto nitrocellulose membranes and stained with antibodies against the following exosomal markers: CD9, CD63, CD81 and TSG101. Figure 2. Immunoblotting analysis of SF-derived exosomes. Exosomes (Exo) purified by immunoaffinity were lysed and protein fraction underwent SDS-PAGE gel electrophoresis under reducing condition. The corresponding IgG fraction was run together with the sample from which they were extracted. The gel was blotted onto nitrocellulose membranes and stained with antibodies against the exosomal markers CD9, CD63, CD81, TSG101 and against RISC complex, calnexin and 58-K which are proteins associated with subcellular compartment. Figure 3. Exosomes isolated by immunoaffinity purification did not express platelet markers. Exosomes were bound by Exo-Flow beads, stained with specific monoclonal antibody for CD81, CD63, CD9, CD61 and CD41a and analysed by flow cytometry. The histograms shown are referred to one representative experiment. Exosome-bound beads (white peak) were compared with beads alone (grey peak) and the percentages of positive beads are reported.
