# Supplementary material for: Characterization of the Proinflammatory Profile of Synovial Fluid-Derived Exosomes of Patients with Osteoarthritis
Source: Mediators Inflamm. 2017 May 28;2017:4814987. doi: 10.1155/2017/4814987 (PMC5467328; doi:10.1155/2017/4814987)
Supplement: Supplementary file 1 — Figure 1. Immunoblotting analysis of SF-derived EVs. EVs isolated by precipitation were lysed and the protein fraction underwent SDS-PAGE gel electrophoresis under reducing condition. The IgG fraction was run together with the sample from which they have been extracted. The gel was blotted onto nitrocellulose membranes and stained with antibodies against the following exosomal markers: CD9, CD63, CD81 and TSG101. Figure 2. Immunoblotting analysis of SF-derived exosomes. Exosomes (Exo) purified by immunoaffinity were lysed and protein fraction underwent SDS-PAGE gel electrophoresis under reducing condition. The corresponding IgG fraction was run together with the sample from which they were extracted. The gel was blotted onto nitrocellulose membranes and stained with antibodies against the exosomal markers CD9, CD63, CD81, TSG101 and against RISC complex, calnexin and 58-K which are proteins associated with subcellular compartment. Figure 3. Exosomes isolated by immunoaffinity purification did not express platelet markers. Exosomes were bound by Exo-Flow beads, stained with specific monoclonal antibody for CD81, CD63, CD9, CD61 and CD41a and analysed by flow cytometry. The histograms shown are referred to one representative experiment. Exosome-bound beads (white peak) were compared with beads alone (grey peak) and the percentages of positive beads are reported. [file 4814987.f1.pdf]

SUPPLEMEMTARY MATERIALS

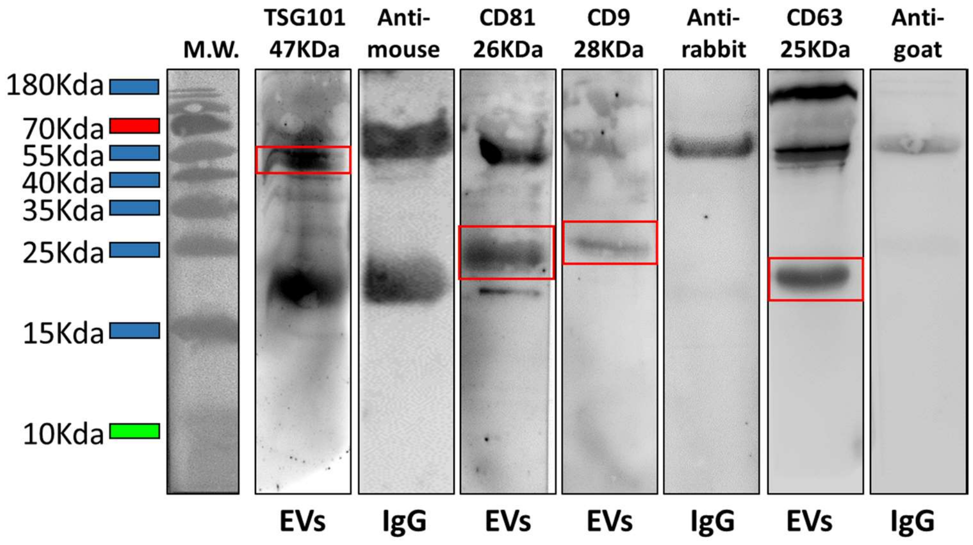

Figure 1. Immunoblotting analysis of SF-derived EVs

EVs isolated by precipitation were lysed and the protein fraction underwent SDS-PAGE gel electrophoresis under reducing condition. The IgG fraction was run together with the sample from which they have been extracted. The gel was blotted onto nitrocellulose membranes and stained with antibodies against the following exosomal markers: CD9, CD63, CD81 and TSG101.

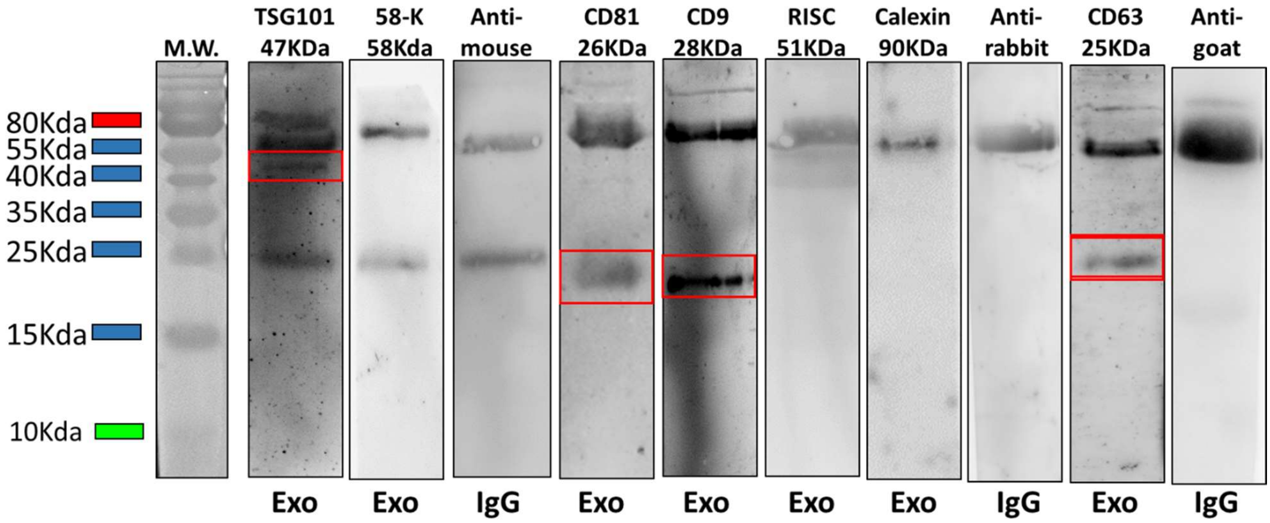

Figure 2. Immunoblotting analysis of SF-derived exosomes

Exosomes (Exo) purified by immunoaffinity were lysed and protein fraction underwent SDS-PAGE gel electrophoresis under reducing condition. The corresponding IgG fraction was run together with the sample from which they were extracted. The gel was blotted onto nitrocellulose membranes and stained with antibodies against the exosomal markers CD9, CD63, CD81, TSG101 and against RISC complex, calnexin and 58-K which are proteins associated with subcellular compartment.

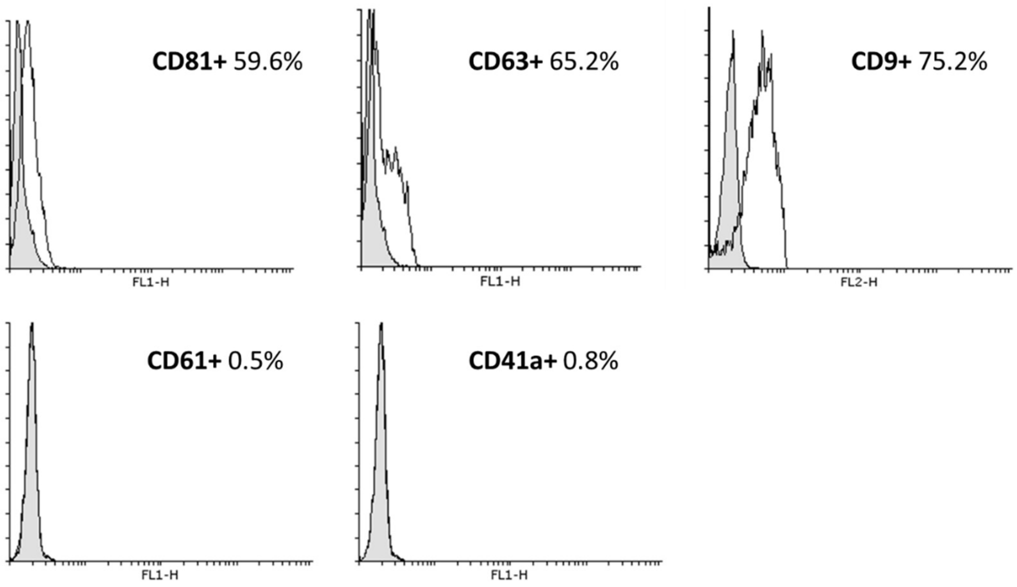

Figure 3. Exosomes isolated by immunoaffinity purification did not express platelet markers
Exosomes were bound by Exo-Flow beads, stained with specific monoclonal antibody for CD81, CD63, CD9, CD61 and CD41a and analysed by flow cytometry.
The histograms shown are referred to one representative experiment. Exosome-bound beads (white peak) were compared with beads alone (grey peak) and the percentages of positive beads are reported.
