# Supplementary figures and images for: Characterization of the Proinflammatory Profile of Synovial Fluid-Derived Exosomes of Patients with Osteoarthritis
Source: Mediators Inflamm. 2017 May 28;2017:4814987. doi: 10.1155/2017/4814987 (PMC5467328; doi:10.1155/2017/4814987)

**
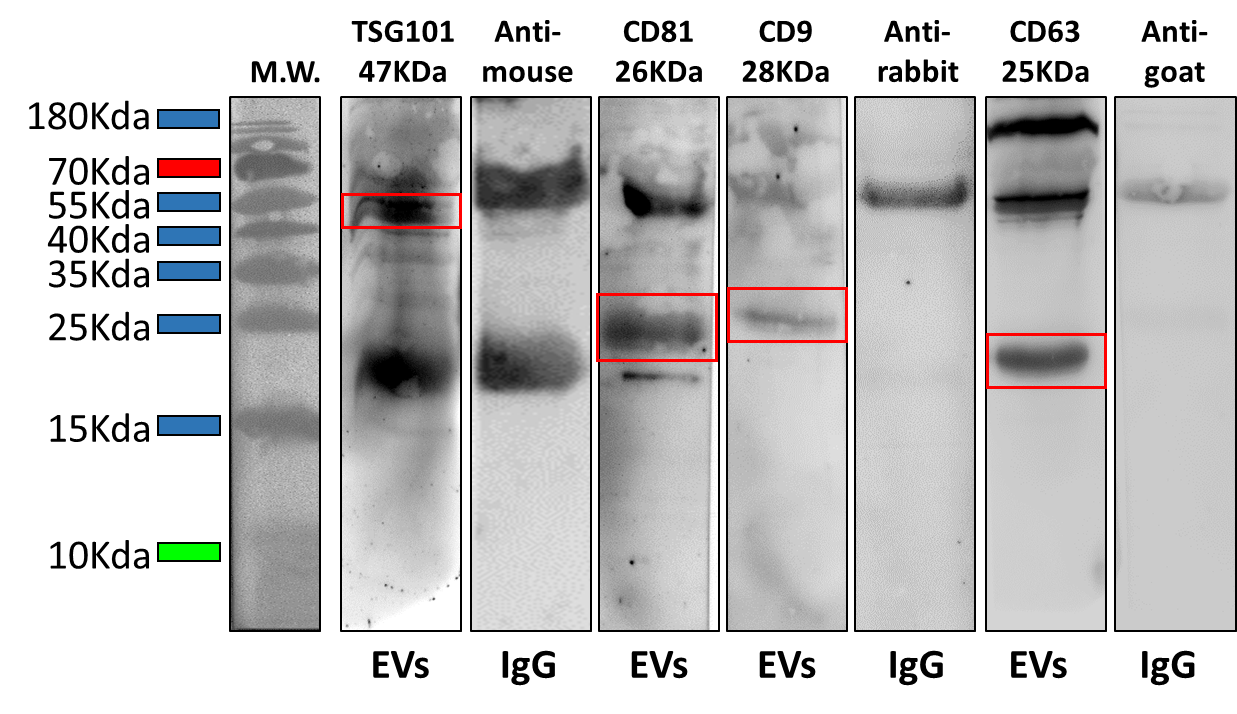
**

Supplement: Supplementary file 2 [file 4814987.f2.docx]

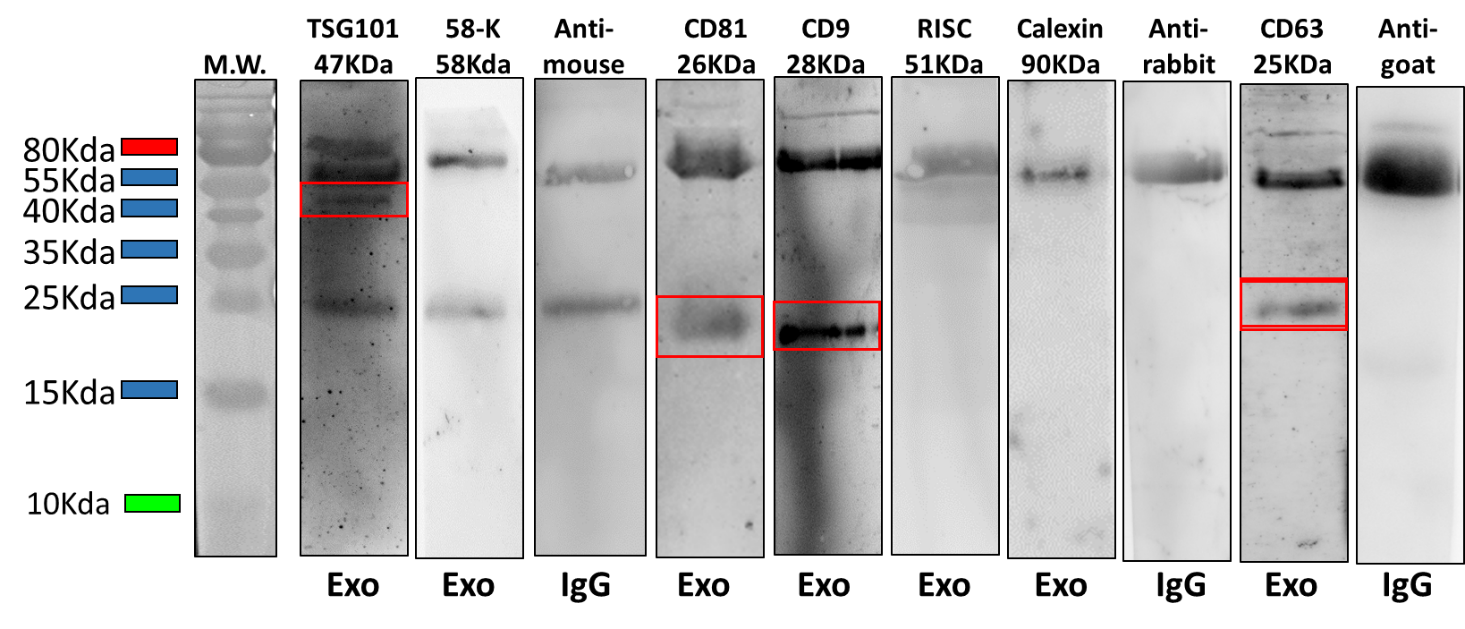

Supplement: Supplementary file 4 [file 4814987.f4.docx]

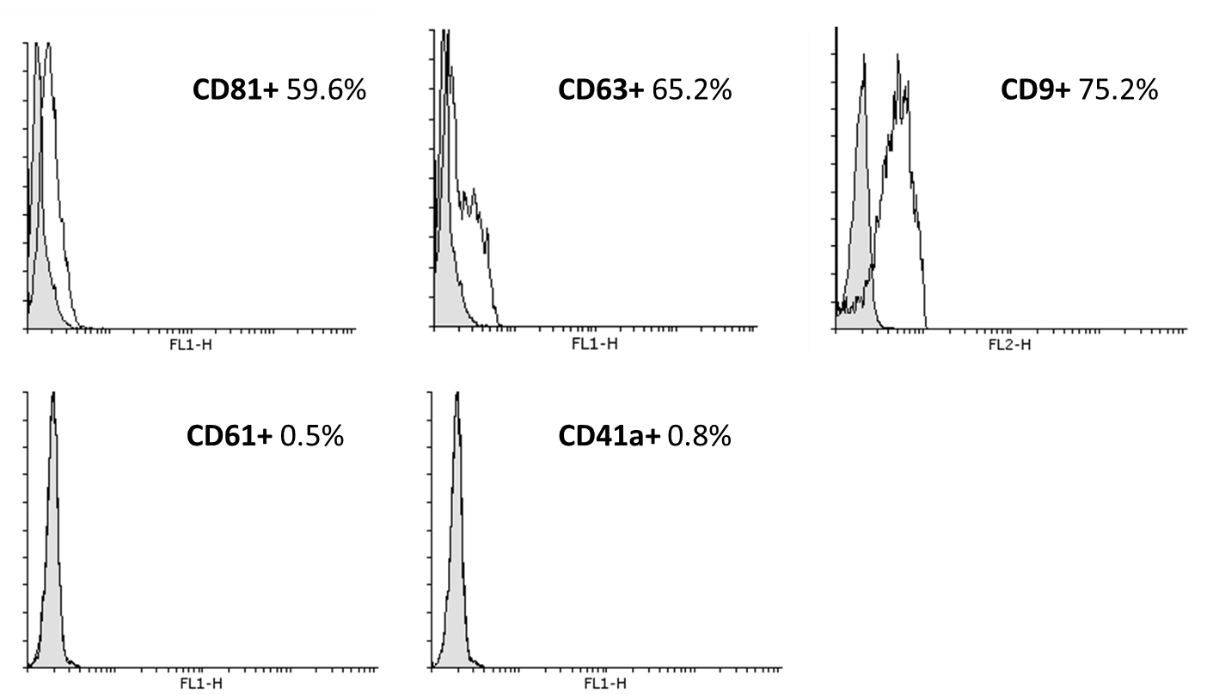

Supplement: Supplementary file 6 [file 4814987.f6.docx]

## Slide 1
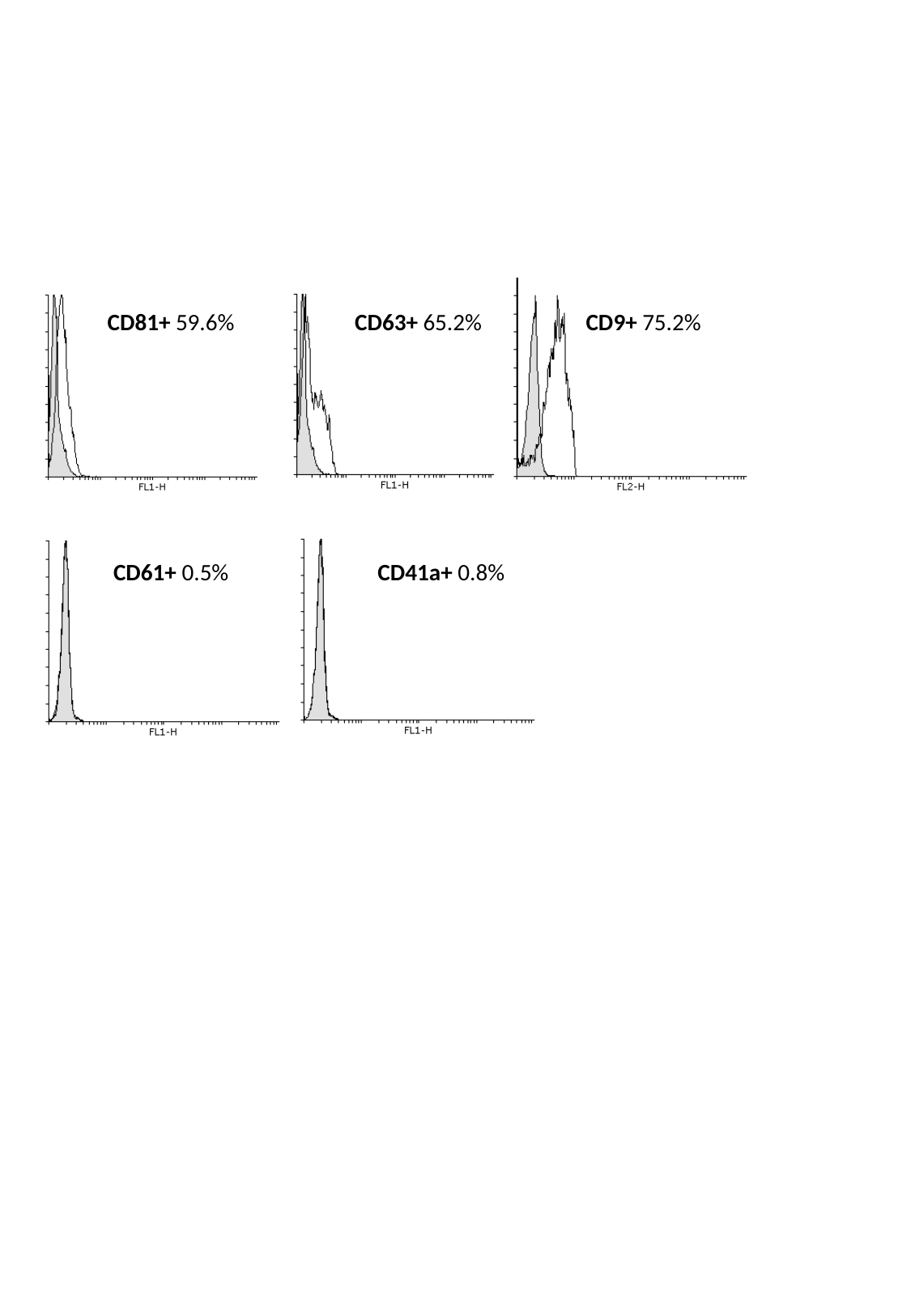

CD81+ 59.6%
CD9+ 75.2%
CD63+ 65.2%
CD41a+ 0.8%
CD61+ 0.5%

Supplement: Supplementary file 7 [file 4814987.f7.pptx]
