# Supplementary material for: Characterization of the Proinflammatory Profile of Synovial Fluid-Derived Exosomes of Patients with Osteoarthritis
Source: Mediators Inflamm. 2017 May 28;2017:4814987. doi: 10.1155/2017/4814987 (PMC5467328; doi:10.1155/2017/4814987)
Supplement: Supplementary file 3 [file 4814987.f3.pptx]

## Slide 1
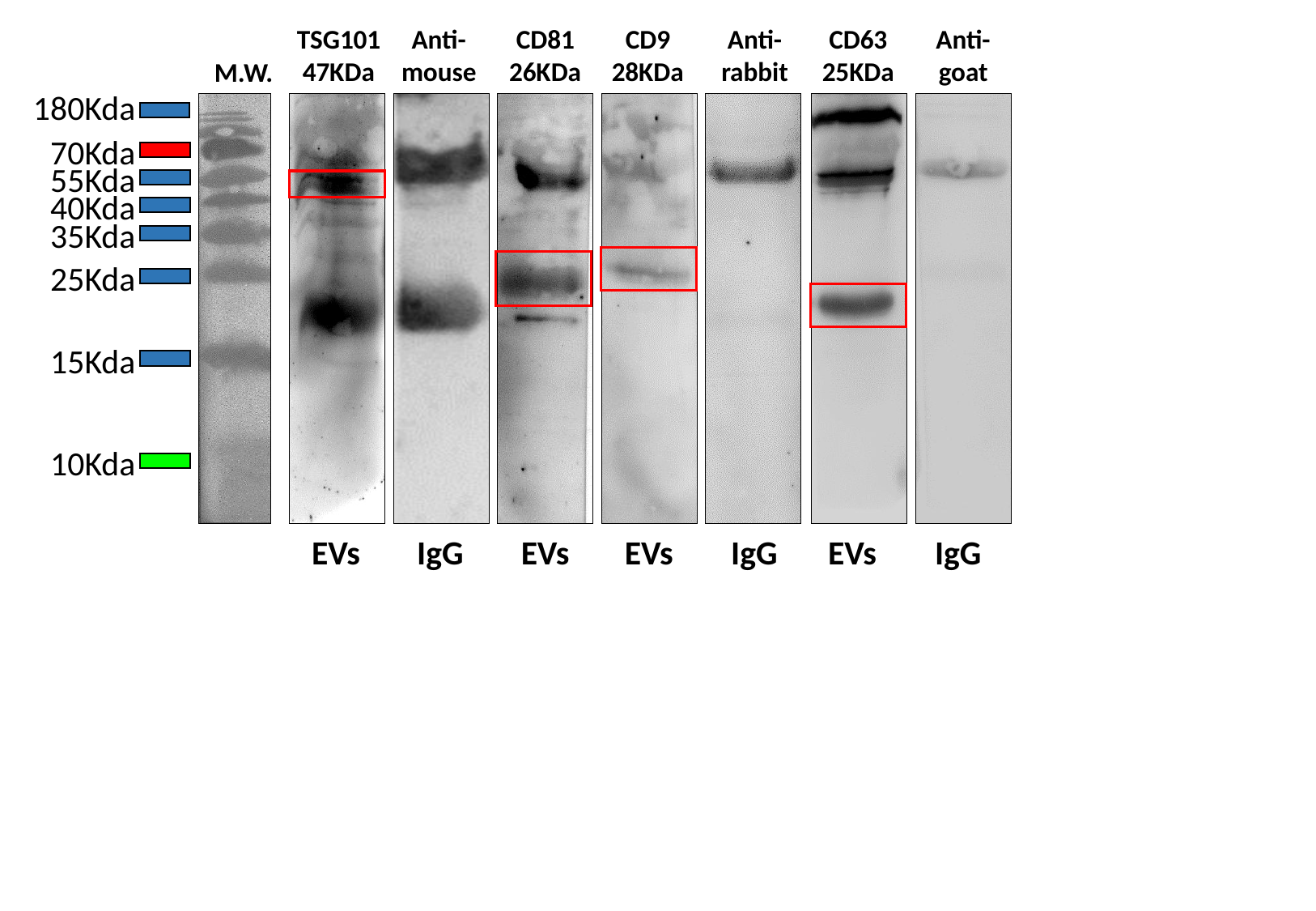

TSG101
47KDa
Anti-
mouse
CD81
26KDa
CD9
28KDa
Anti-
rabbit
CD63
25KDa
Anti-
goat
M.W.
180Kda
70Kda
55Kda
40Kda
35Kda
25Kda
15Kda
10Kda
EVs
IgG
EVs
EVs
IgG
EVs
IgG
