# Supplementary material for: Characterization of the Proinflammatory Profile of Synovial Fluid-Derived Exosomes of Patients with Osteoarthritis
Source: Mediators Inflamm. 2017 May 28;2017:4814987. doi: 10.1155/2017/4814987 (PMC5467328; doi:10.1155/2017/4814987)
Supplement: Supplementary file 5 [file 4814987.f5.pptx]

## Slide 1
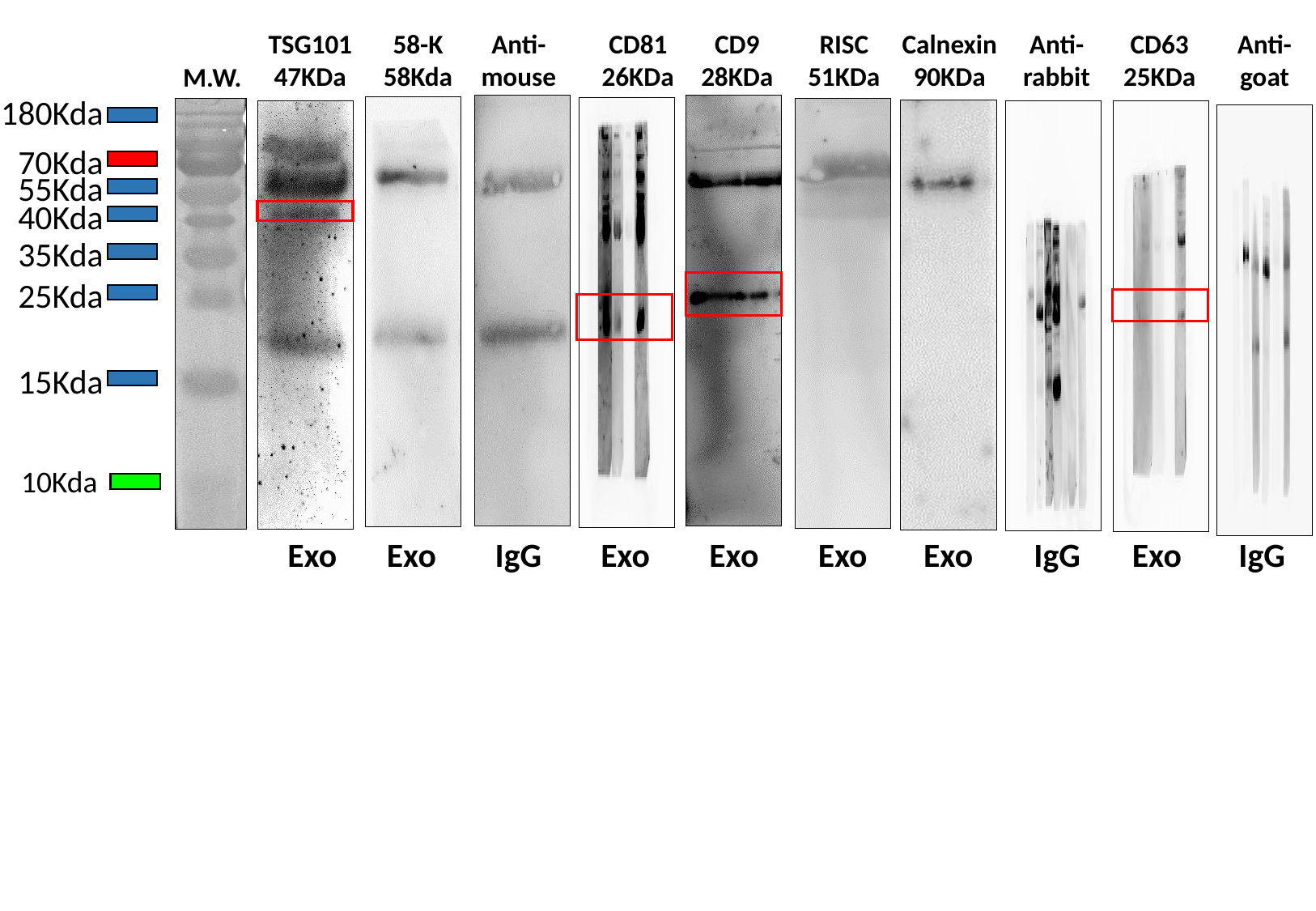

TSG101
47KDa
58-K
58Kda
Anti-
mouse
CD81
26KDa
CD9
28KDa
RISC
51KDa
Calnexin
90KDa
Anti-
rabbit
CD63
25KDa
Anti-
goat
M.W.
180Kda
70Kda
55Kda
40Kda
35Kda
25Kda
15Kda
10Kda
Exo
Exo
IgG
Exo
Exo
Exo
Exo
IgG
Exo
IgG
